# Bilayer Heterogeneous Cation Exchange Membrane with Polyaniline Modified Homogeneous Layer: Preparation and Electrotransport Properties

**DOI:** 10.3390/membranes13100829

**Published:** 2023-10-11

**Authors:** Natalia Loza, Irina Falina, Natalia Kutenko, Svetlana Shkirskaya, Julia Loza, Natalia Kononenko

**Affiliations:** Physical Chemistry Department, Faculty of Chemistry and High Technologies, Kuban State University, 350040 Krasnodar, Russia; nata_loza@mail.ru (N.L.); natalakutenko818@gmail.com (N.K.); shkirskaya@mail.ru (S.S.); julialoza@list.ru (J.L.); kononenk@chem.kubsu.ru (N.K.)

**Keywords:** cation exchange membrane, modification, polyaniline, bilayer membrane, structure, conductivity, diffusion permeability, selectivity, transport number, current–voltage curve

## Abstract

A bilayer membrane based on a heterogenous cation exchange membrane with a homogeneous cation exchange layer and a polyaniline on its surface is prepared. The intercalation of polyaniline into the membrane with a homogeneous cation exchange layer is performed by oxidative polymerization of aniline. The influence of the homogeneous cation exchange layer and the polyaniline on the structure, conductivity, diffusion permeability, selectivity, and current–voltage curve of the heterogeneous cation exchange membrane is established. Membrane properties are studied in the HCl, NaCl, and CaCl_2_ solutions. The homogeneous cation exchange layer has a negligible effect on the transport properties of the initial heterogeneous membrane. The polyaniline synthesis leads to a decrease in the macropore volume in the membrane structure, conductivity, and diffusion permeability. The counterion transport number in the bilayer membrane is significantly reduced in a solution of calcium chloride and practically does not change in sodium chloride and hydrochloric acid. In addition, the asymmetry of the diffusion permeability and shape of current–voltage curve depending on the orientation of the membrane surface to the flux of electrolyte or counterion are found.

## 1. Introduction

Electrodialysis allows for a number of important processes, such as separation and concentration of electrolyte solutions, extraction of precious and non-ferrous metal salts, conditioning of food raw materials and products, separation of organic acids from mixed solutions, removal and recovery of nutrients from wastewater [1,2,3,4,5,6,7,8,9], etc. The main element of the electrodialyzer is ion exchange membranes (IEMs). A set of electrotransport and structure properties of IEMs ensures the efficiency of electrodialysis [10]. Thus, high conductivity and selectivity provide high current efficiency and low energy consumption in the process. The low electroosmotic permeability of the membranes allows for efficient electrolyte solution concentration [11]. Available commercial membranes, despite their variety [12,13,14], cannot fully satisfy the needs of electrodialysis processes. Therefore, a large number of articles is dedicated to developing new types of IEMs or modifying the existing ones [12,13,15,16,17].

One of the most challenging problems in the electrodialysis conditioning of wastewater is not only to increase or decrease concentrations of all the present electrolytes but to separate them. For example, the separation of lithium salts from seawater [18,19], recovery of heavy metal salts and acids from industrial wastewater [3,20,21], etc. For a successful separation of multiply charged metal salts from singly charged ones or acids, cation exchange membranes are used, selectively transporting singly charged cations and preventing the transport of multiply charged ones [3,22]. Monovalent cation exchange membrane permselectivity is provided by different physical phenomena such as the pore-size sieving effect, stronger electrostatic repulsion between the anion exchange surface layer and multivalent cations than monovalent cations, and the hydration energy difference caused by the different Gibbs hydration energy of cations [23,24,25].

The creation of the polyaniline (PANI) layer on one of the surfaces of the cation exchange membrane leads to monovalent permselectivity due to the positive charge of the nitrogen [26,27]. The PANI layer on one of the surfaces of the commercial cation exchange membranes Neosepta CMX or CM-1 and Nafion has provided the permselectivity between singly and doubly charged cations such as Na^+^/Mg^2+^, Na^+^/Ca^2+^, Na^+^/Sr^2+^, Na^+^/Ba^2+^ [23,28] H^+^/Zn^2+^ or H^+^/Cu^2+^ [27,29,30,31,32]. Transport of singly charged ions through membranes after their modification has increased by 10–12 times compared to doubly charged ones. The efficiency of Na^+^/Ca^2+^, Na^+^/Cu^2+^, Na^+^/Zn^2+^, and Na^+^/Al^3+^ separation is shown when using experimental membranes single-face modified with PANI experimental membranes [33,34].

The homogeneous or interpolymer cation exchange membranes are used in these studies. The homogeneous membrane Nafion consists of perfluorovinyl ether pendant side chains terminated by sulfonic or carboxylic ionic functional groups. The homogeneous membrane Neosepta CMX consists of sulfonated polystyrene crosslinked with divinylbenzene and polyvinylchloride as a support [35]. The basic polymer matrix of an experimental membrane is an interpolymer of polyethylene and styrene–divinyl benzene copolymer. Their structure is much more homogeneous than that of the conventional heterogeneous membranes widely used in electrodialysis. This factor is supposed to have a significant impact on the peculiarities of ion transport in such electromembrane systems.

The methods of preparation and the electrotransport properties of composites based on PANI and ion-exchange membranes are described in a large number of studies, which are associated with a wide range of applications for such materials. The prospects of using such materials in various sensor systems [36,37,38,39,40,41] and methanol fuel cells and redox flow batteries [42,43,44,45,46,47,48] are most often considered.

The most common methods of membrane modification with PANI are in situ oxidative polymerization [26,27,29,33,34,36,37,39,45,46,49], electrochemical synthesis of PANI [30,44], and PANI suspension or solution injection [38,47,50,51,52]. In order to obtain membranes single-face modified with PANI, the synthesis is carried out under the condition of successive diffusion of aniline and oxidizer through the membrane to water. At the same time, it is found that the modification conditions, such as the concentration of the oxidizer and the time of contact with monomer and oxidizer solutions, affect the distribution of the modifier in the membrane and the transport characteristics of the obtained composites. Thus, the increase of the aniline polymerization time or the increase of its content leads to the appearance of PANI chains not only on the surface [53], but also in the volume of the membrane [26,29]. A decrease in the specific conductivity and diffusion permeability of the obtained composites is also observed [29,47,54,55,56]. However, in the case of a low content of the modifier in the membrane, an increase in its specific conductivity and diffusion permeability might be observed, which is explained by the expansion of the pore space of the membrane [37,55]. In some cases, an increase in the specific conductivity of PANI-modified membranes compared to the original ones is also observed at relatively high modifier contents [33]. So, it is obvious that the content of the modifier, its distribution in the membrane matrix, and the peculiarities of the initial membrane structure have a significant impact on the properties of the composite.

In our previous studies, it is shown that surface modification of homogeneous perfluorinated MF-4SK and heterogeneous MK-40 membranes with sulfonic acid groups leads to a decrease in their diffusion and electroosmotic permeability, as well as specific conductivity. However, for homogeneous membranes, these effects are more pronounced [57,58,59,60].

A study of the competitive transfer of singly and doubly charged cations (Na^+^/Ca^2+^, H^+^/Ca^2+^, H^+^/Ni^2+^) in electrodialysis using the initial and surface-modified with PANI homogeneous MF-4SK membranes and heterogeneous MK-40 membranes is carried out in the studies [61,62]. Applying the composites based on homogeneous membranes has proven effective for the separation of singly and doubly charged ions, as well as the selective concentration of sulfuric acid with its simultaneous separation from a mixed sulfuric acid and nickel sulfate solution. However, composites based on a heterogeneous membrane and PANI demonstrate an insignificant increase in specific selectivity to singly charged ions compared to the initial membranes.

The peculiarities of ion transfer through cation exchange heterogeneous and homogeneous membranes surface-modified with PANI are due to their different structure and location of the modifier. Ion exchange membranes are not typical porous bodies [63]. The system of cavities and channels in the phase of the ion-exchange membrane, through which the transfer of ions and solvent molecules occurs, is formed as a result of its swelling in the solvent [64,65]. At the same time, macropores appear alongside micro- and mesopores. According to the method of reference contact porosimetry, pores with an effective radius of up to 250 nm are present in the structure of homogeneous membranes, while macropores with an effective radius of up to 1000 nm are found in heterogeneous membranes [63].

**Figure 1:**
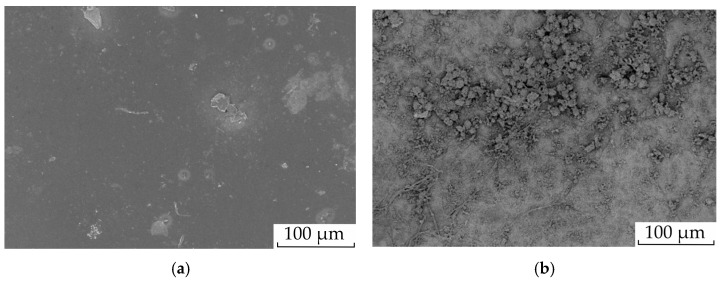
Surface SEM images of initial membrane MF-4SK (**a**) and modified one (**b**). Adopted from [59].

The surface of a homogeneous membrane is rather smooth (Figure 1a). The crystallites about 4 nm in size appearing in the perfluorinated membrane structure [66] do not interfere with aniline polymerization. As such, the polyaniline is formed on the entire surface of the membrane. The SEM image clearly shows that the entire modified membrane surface is covered with globules 4–6 µm in size and filamentous formations 0.7–2 µm thick PANI (Figure 1b) [59].

**Figure 2:**
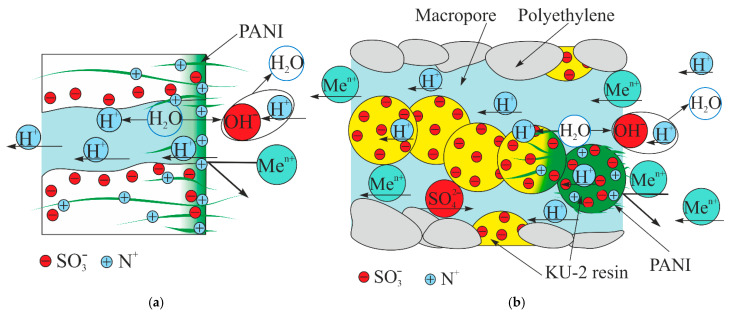
Scheme of the transfer of hydrogen and doubly charged cations through the surface-modified homogeneous MF-4SK/PANI membrane (**a**) and the heterogeneous MK-40 membrane (**b**). PANI is marked green.

During electrodialysis, doubly charged cations are repelled by the positively charged PANI layer, while singly charged cations pass through it. Thus, the separation of singly and doubly charged cations occurs (Figure 2a). If a hydrogen cation acts as a singly charged cation, then one more transfer mechanism might be assumed. Positively charged amino groups of PANI and sulfonic acid groups of the membrane form bipolar contacts. Catalytic water splitting occurs on them, producing protons, which then transfer to the concentration chamber.

**Figure 3:**
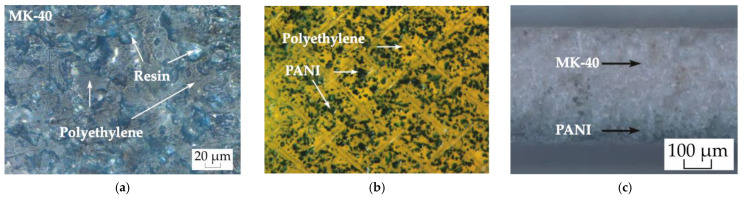
Image of the surface of the initial MK-40 membrane (**a**), the modified surface (**b**), and the cross-section of the MK-40/PANI composite (**c**) [59].

The surface of a heterogeneous membrane consists of alternating conductive sections, which are particles of a cation exchange resin and non-conductive polyethylene. In the process of swelling, the grain of the ion-exchange resin significantly changes its size. The polyethylene that surrounds the ion exchange resin grain does not absorb water and does not change its dimensions. As a result, large defects are formed at the junction of polyethylene and grains of the ion exchange resin during swelling. These defects are clearly visible on SEM images of the surface of swollen and then dried membranes [67,68]. The sizes of grain outlets of the cation exchange resin on the surface vary in a fairly wide range from 5 to 30 µm, and the distance between them does not exceed 50 µm. Such sizes of inhomogeneities on the surface of a heterogeneous membrane significantly exceed those on the surface of a homogeneous membrane. At the same time, polyethylene occupies most of the heterogeneous membrane surface (Figure 3a). The proportion of the conductive surface of the swollen MK-40 membrane is only about 0.2 [67,69]. Similarly, large cavities are formed in the volume of the membrane around the grains of the ion exchange resin. In general, a network of such large pores forms part of the channels with non-selective transfer in the membrane [63]. However, the grains of the ion exchange resin do not contain macropores [70].

PANI forms on the grains of the cation exchange resin and is absent on polyethylene and reinforcing mesh (Figure 3b,c) [59,71]. Also, as in the case of MF-4SK/PANI, there is a gradient distribution of the modifier from the surface into the volume of the membrane (Figure 3c). However, unlike MF-4SK/PANI, there is a discontinuous modifier layer on the surface of the heterogeneous membrane. Hence, the transport of ions through macropores will proceed similarly to the initial membrane and the separation of singly and doubly charged ions will only occur in selective channels directly in the cation exchange resin (Figure 2b).

**Figure 4:**
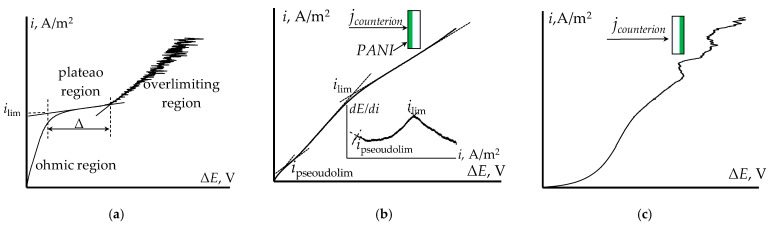
An illustrative conventional ion exchange membrane CVC (**a**) and illustrative CVCs of the MF-4SK/PANI membrane with surface distribution of modifier for the orientation I (PANI layer on the counterion flux) (**b**) and orientation II (initial membrane surface on the counterion flux) (**c**).

Current–voltage curves of the membranes are widely used for studying their electrotransport behavior under the concentration polarization condition close to electrodialysis [17,72,73,74,75,76]. A typical current–voltage curve (CVC) of IEM consists of three specific regions (Figure 4a) [77,78,79]. The first one is the ohmic region corresponding to the linear increase in the potential drop across the membrane (PD) with the increase of current density. The counterion transport numbers through the membrane are significantly higher compared to the solution, leading to the development of concentration polarization in the depleted diffusion layer. Counterion concentration is gradually decreasing and tends to be zero. The current density corresponding to this state is called the limiting current density (*i*_lim_). It is reflected in the CVC by the formation of the limiting current plateau. Further intensification of the concentration polarization leads to the catalytic water splitting and the appearance of new mechanisms of counterion transport from the solution, mainly electroconvection [77,80,81,82,83]. A new, approximately linear region of increasing current density and PD appears on the CVC. In this region of a CVC corresponding to the overlimiting state, there are PD oscillations present, related to the appearance and evolution of electroconventive vortices near the membrane surface, as well as water splitting [83,84].

The main parameters of the CVC are limiting current density, ohmic and overlimiting region slopes, limiting current plateau length (Δ), and potentials of transiting the system to the limiting and overlimiting states. The CVC is dependent on the peculiarities of the membrane surface [85,86,87,88,89]. The geometrical or electrical heterogeneity of the surface has a great impact on the electroconvection development and CVC parameters [83,90,91,92,93,94,95,96]. As PANI mainly affects the microstructure of the surface, a change in the CVC of the modified membrane is expected.

The analysis of the initial and PANI-modified homogeneous membranes’ CVCs showed that the appearance of PANI on one of the surfaces leads to asymmetry in the CVC [61,97,98]. On the CVC measured with the membrane oriented with the modified surface towards the counterion flow (orientation I), a decrease in the limiting current density, a decrease in the specific conductivity in the ohmic region, and an increase in the limiting current plateau is observed, compared to the initial membrane. For the modified membrane CVC with orientation I and low PD (up to 0.05 V), a pseudolimiting current (*i*_pseudolim_) is found (Figure 4b). It is due to a depletion of a bipolar boundary between the positively charged PANI layer and the negatively charged initial membrane matrix. This is reflected in the differential CVC of the surface-modified membrane by the formation of two extrema (Figure 4b). The CVC measured with the membrane oriented with the unmodified surface towards the counter-ion flow (orientation II) has an unusual shape, lacking any of the characteristic regions. However, after achieving a certain current density and PD, characteristic oscillations of current density and PD corresponding to the overlimiting state appear (Figure 4c).

The small impact of PANI on ion transport in a composite based on a heterogeneous membrane is confirmed by a slight change in the shape and parameters of the CVCs of these composites compared to the initial membranes. There is no “pseudo-limiting” current on the CVC of MK-40/PANI and there is practically no asymmetry of the curve depending on the orientation of the membrane to the flux of counterions [59,61]. Thus, the CVC can be used as a test signal to assess the presence of specific membrane selectivity to singly charged cations.

It has been shown that an increase in the time of PANI synthesis on the surface of heterogeneous membranes up to 4 h leads to a decrease in the proportion of macropores with an effective radius of more than 100 nm [99]. However, these changes do not strongly affect the specific electrical conductivity, diffusion permeability, and the shape of the CVC of the composites. So, an increase in the amount of PANI on the surface does not allow for the creation of a sufficiently homogeneous barrier layer of the modifier.

In the present study, it is planned to change the microstructure of a heterogeneous cation exchange membrane surface by depositing a thin film of a homogeneous cation exchange polymer on it, followed by the formation of a barrier layer of the modifier. This should provide conditions for the uniform formation of a polyaniline layer on the membrane surface. Comprehensive characterization of the obtained materials, including the study of structural characteristics by the method of reference contact porosimetry, determination of specific conductivity, diffusion permeability, and CVCs in solutions of sodium chloride, calcium, and hydrochloric acid will allow us to evaluate the prospects for using the obtained materials in electrodialysis.

## 2. Materials and Methods

### 2.1. Cation Exchange Membrane and Preparation of the Bilayer PANI-Modified Membrane

The MK-40 membrane (Ltd. “Innovative Enterprise Shchekinoazot”, Tula Region, Russia) is used as the initial membrane. It is a heterogeneous material, consisting of KU-2 cation exchange resin, polyethylene, and reinforcing polyamide mesh. KU-2 cation exchange resin is polystyrene crosslinked by divinylbenzene containing the sulfonic acid groups as fixed ions.

The membranes were conditioned according to the standard procedure. The membranes were placed in ethanol for 6 h. Next, the membranes were sequentially placed for 24 h in sodium chloride solutions with concentrations of 312 g/L (saturated solution), 100 g/L, and 30 g/L. After that, the membranes were placed in deionized water for 48 h. Then, the membranes were converted into the form of hydrogen cations by placing them in a 10% hydrochloric acid solution for 48 h. Lastly, the membranes were washed with deionized water until neutral with methyl orange after a day of contact of the membranes with a portion of deionized water.

In the first stage, samples of MK-40 membranes with a layer of MF-4SK on one of the surfaces were obtained. For this, a dispersion of LF-4SK (10 wt. %) (JSC Plastpolymer, St. Petersburg, Russia) in isopropyl alcohol was used. LF-4SK is the perfluorosulfonic acid. The ion exchange capacity of the LF-4SK is 0.98 mmol-eq per g of dry polymer. Before applying to the membrane, LF-4SK was mixed with glacial acetic acid to improve adhesion. The membrane was pre-dried at room temperature for 48 h. The amount of the modifier was 2.5 mL of a 10% dispersion of LF-4SK per 1 dm^2^ of the membrane. After the applied MF-4SK layer dried, the membranes were conditioned similarly to the initial ones, except for placing them in ethyl alcohol. The MK-40 sample with an MF-4SK layer on one surface is marked MK-40/MF-4SK.

The conversion of membranes into the required ionic form was carried out by placing the samples in a 10% solution of the corresponding electrolyte, followed by washing with deionized water from the non-exchange absorbed solution.

**Figure 5:**
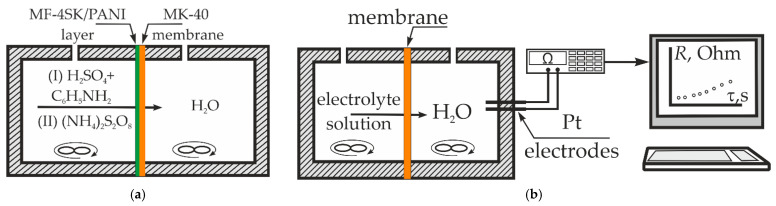
The scheme of PANI synthesis on the membrane surface (**a**) and the experimental setup for the diffusion permeability measurement (**b**).

The synthesis of PANI on the surface of the MK-40/MF-4SK membrane was carried out under the conditions of successive diffusion of a monomer solution (0.1 M aniline in 0.05 M sulfuric acid) and an oxidizer solution (0.1 M ammonium persulfate) through the membrane into water (Figure 5). In the first stage, the membrane is in contact with 0.1 M aniline solution in 0.05 M sulfuric acid with the surface containing MF-4SK layer. Anilinium cations transfer to the membrane phase via the ion-exchange and diffusion mechanisms. In the second stage, monomer solution is switched to the oxidizer solution. PANI forms in this stage. The contact time with the monomer and oxidizer solution varied: 2 and 4 h for each sample. The membrane surface with the MF-4SK film was turned towards the monomer and oxidant solutions, which ensured the formation of PANI on the desired membrane surface. In the first stage of modification, the membrane was saturated with anilinium ions, and in the second stage, it was polymerized under the action of ammonium persulfate. Since the persulfate anion is a co-ion with respect to the cation exchange membrane, its penetration into the membrane phase is difficult according to the Donnan exclusion. It is assumed that under such conditions, PANI was formed predominantly on the membrane surface. The time of PANI synthesis was taken as the time of contact of the membrane with the oxidizer solution. The modified samples were designated as MK-40/MF-4SK/PANI-2 and MK-40/MF-4SK/PANI-4, where 2 and 4 were the time of PANI synthesis in hours.

After the modification, the membrane was placed in 0.5 M sulfuric acid for 24 h to remove the remaining monomer and then washed with deionized water. The modification of the MK-40 membrane with the MF-4SK film and the synthesis of PANI did not lead to a change in its ion exchange capacity (Q) (Table 1).

### 2.2. Diffusion Permeability, Conductivity, and Current–Voltage Curves

The membranes were brought into equilibrium with an electrolyte solution before measuring the transport characteristics. To achieve this state, the membrane was placed in an electrolyte solution with the required concentration. Portions of the solution were changed 3–4 times on the first day. Further, the membrane was placed in one portion of the solution for 24 h, after which the electrical conductivity of the solution was measured. The criterion for the onset of equilibrium was the equality of the electrical conductivity of the solution before and after contact with the membrane. If equilibrium was not reached, the procedure was repeated.

The techniques thoroughly described in [61,100] were used in order to determine the diffusion permeability coefficient, specific conductivity, and current–voltage curves (CVCs) measurements. All experiments were carried out at the constant temperature of 25 °C and were performed at least 3 times. The diffusion permeability and CVC measurements were performed for both membrane orientations when the modified or initial membrane surface was placed toward electrolyte solution or counter-ion flux. The average values, standard deviations, confidence intervals, and absolute errors were calculated for all experimental data using standard features of Microsoft Excel.

The diffusion permeability of membranes was obtained using a non-flow two-chamber cell (Figure 5b). The test was membrane separated into two chambers. The first chamber contained a 0.5 M NaCl solution, whereas deionized water and platinum electrodes for the resistance measurement were in the second one. The solutions in both chambers were stirred. The value of the diffusion permeability coefficient (*P*) was calculated by the formula

(1) $$P=\frac{V}{S}K\frac{d(\frac{1}{R_{s}})}{d\tau }\frac{l}{C}$$

where *V* was the chamber volume (1 × 10^−7^ L); *S* was the membrane area (2.1 × 10^−3^ m^2^); *R_s_* was the resistance in the second chamber; *τ* was the time of experiment; *C* was the electrolyte concentration in the first chamber; and *K* was the constant of the diffusion cell that depends on the geometry of the electrodes and the electrolyte nature. The *K* value was obtained as a slope of dependence in coordinates C−(1Rs).

**Figure 6:**
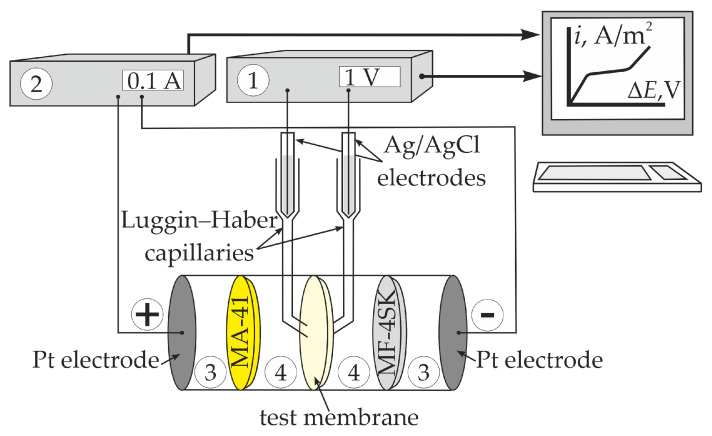
The experimental setup for the membrane CVC measurement: 1 was the Keithley 2701 Ethernet Multimeter/Data Acquisition System (Keithley Instruments, Inc., Cleveland, OH, USA); 2 was the Keithley 2420 SourceMeter (Keithley Instruments, Inc., Cleveland, OH, USA); 3 were the electrode chambers; and 4 were the test membrane chambers.

The CVC of the membrane was measured using cell consisting of four chambers separated by vertically fixed membranes (Figure 6). Auxiliary MA-41 and MF-4SK membranes were placed between Pt electrodes and the test membrane. The MA-41 membrane (Ltd. “Innovative Enterprise Shchekinoazot”, Tula Region, Russia) was the heterogeneous anion exchange membrane containing the quaternary amino groups. The electrolyte solution circulated through each chamber from individual tanks at a flow rate of 14 mL/min. The CVC was measured at a scanning rate of 5 × 10^−5^ A/s.

The conductivity of membrane (*k*) was obtained from the active part of the membrane AC resistance measured by mercury contact method. The conductivity was calculated by formula

(2) $$k=\frac{l}{R_{m}\cdot S}$$

where *R_m_* was the membrane resistance. The resistance was measured using the potentiostat–galvanostat Autolab PGSTAT302N equipped with FRA-32 impedance unit (Metrohm Autolab B.V., Utrecht, The Netherlands).

### 2.3. Standard Contact Porosimetry and Optical Microscopy

The photographs of the cross sections of the wet modified membranes samples were obtained with the Altami BIO-2 optical microscope equipped with a UCMOSO5100KPA digital ocular USB camera.

The structure of the wet membranes was studied with the method of standard contact porosimetry based on the laws of capillary equilibrium [63,101]. This method allows for the measurement of pore volume distribution by pore radii in a wide range of radii for the studied membrane, total water content (*V*_0_, cm^3^/g), and the total area of the internal specific surface (S, m^2^/g). Also, the volume of unselective macropores (*V*_macro_) and the volume of micro- and mesopores (*V*_micro_) having nearly ideal selectivity are defined. The boundary between selective and non-selective pores is at a pore radius of 25 nm [63]. Device for standard contact porosimetry measurements and determination of pore structure characteristics пoдрoбнo oписаны здесь are thoroughly described in [63].

## 3. Results and Discussion

### 3.1. Study of the Membrane Structure

Since the initial membrane is yellow and the emerging modifier is green, its formation on the surface or in the volume of the membrane can be assessed visually. For samples with a synthesis time of 2 h, it is clearly seen that PANI is formed on the surface and slightly penetrates into the volume. However, for the sample with a modification time of 4 h, separate light green areas are visible on the conditionally unmodified side, indicating the formation of PANI throughout the membrane volume. As a result of the long contact time of the membrane with the ammonium persulfate solution, it diffuses into the volume of the membrane. Since aniline is present in the entire volume of the membrane, the appearance of a sufficient amount of the oxidizer leads to the formation of PANI in the volume of the membrane. In this case, the modifier has a gradient distribution in volume: the main part is formed in the surface layers, which then decreases towards the opposite side of the membrane. This makes the further increase of the aniline polymerization time under the given synthesis conditions inexpedient.

**Figure 7:**
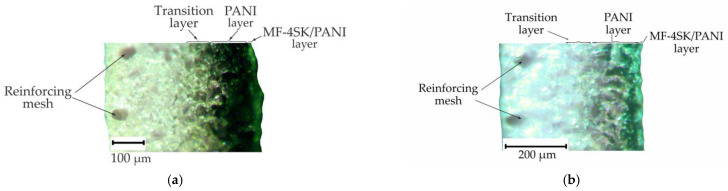
Cross-sectional images of the MK-40/MF-4SK/PANI-2 membrane (**a**) and MK-40/MF-4SK/PANI-4 membrane (**b**).

To assess the thickness of the modified part of the cation exchange membranes, microphotographs of composite cross-sections were made (Figure 7). Three parts of the modified layer are conditionally distinguished. There is an MF-4SK/PANI layer with a thickness of about 20 µm on the surface of the membranes. The same thickness of this layer is due to the fact that the same amount of LF-4SK has been applied. Next is an intensely colored layer in which all resin grains contain PANI. This layer is designated in Figure 5 as the PANI layer. A less-intensely colored layer in which PANI has a gradient distribution is labeled as the transition layer. The thickness of the second layer does not depend on the modification time and is about 140 µm for both samples. However, the thickness of the transition layer increases with the increasing composite synthesis time from 90 µm for the MK-40/MF-4SK/PANI-2 sample to 140 µm for the MK-40/MF-4SK/PANI-4 sample. Thus, 2 h of synthesis is sufficient to modify almost all grains of the cation exchange resin to a depth of 140 μm, which is about 25% of the entire membrane thickness. A further increase in the synthesis time under these conditions leads to an increase in the penetration depth of the modifier. However, this is not accompanied by an increase in the amount of PANI on the surface and in the surface layer.

**Figure 8:**
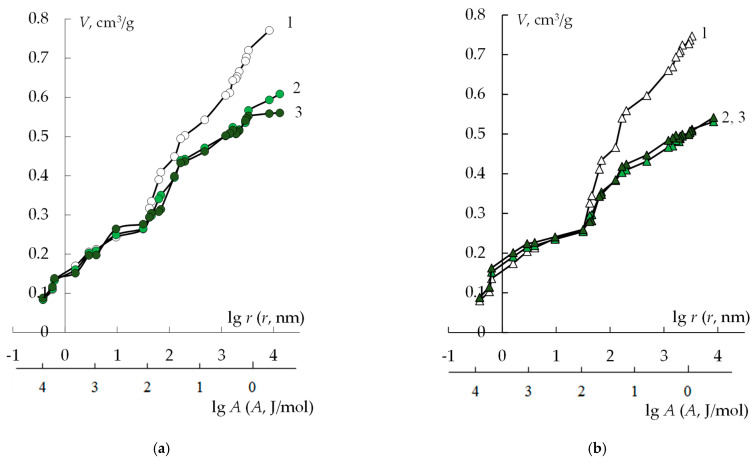
Integral functions of water distribution on the water binding energy and the effective pore radii for the MK-40/MF-4SK (1), MK-40/MF-4SK/PANI-2 (2), and MK-40/MF-4SK/PANI-4 (3) membranes in H^+^-form (**a**) and Ca^2+^-form (**b**).

Figure 8 and Table 2 show the results of studying the structure of the original MK-40/MF-4SK and modified membranes in various ionic forms. In general, the transition from H^+^ to Ca^2+^ form leads to a decrease in the maximum water content of all the studied membranes. This is due to a smaller number of cations in the membrane phase, resulting in a decrease in the total amount of bounded water.

The maximum water content of the MK-40 membrane decreases after the synthesis of PANI by about 25%, regardless of the time of the sample modification. The proportion of gel pores that provide membrane selectivity somewhat increases as a result of modification. At the same time, the proportion of macropores in the membrane structure decreases. This might be explained by the fact that PANI chains are located in macropores, displacing water from them, which leads to a decrease in the maximum water content and a decrease in the effective pore size due to the fact that part of the space in them is occupied by the modifier.

### 3.2. Diffusion Permeability and Conductivity of the Membranes

**Figure 9:**
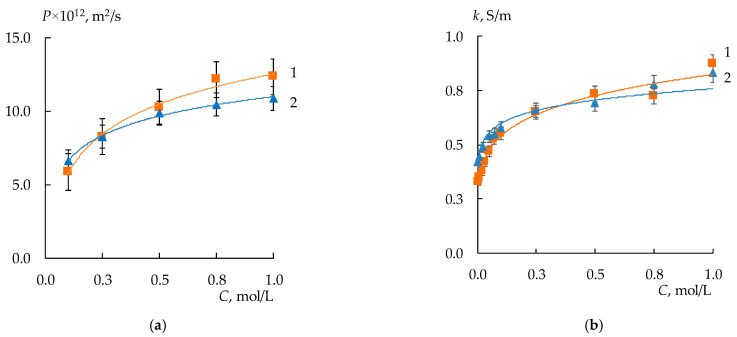
Diffusion permeability (DP) concentration dependencies (**a**) and conductivity concentration dependencies (**b**) for the initial MK-40 membrane (curves 1) and MK-40/MF-4SK one (curves 2) in NaCl solutions.

The deposition of an MF-4SK film on the surface of a heterogeneous MK-40 membrane does not significantly affect the electrotransport properties of the membrane (Figure 9a,b). However, there is a decrease in the diffusion permeability of the MK-40/MF-4SK membrane compared to the diffusion permeability of the initial MK-40 membrane. This is especially noticeable in more concentrated solutions. The decrease is 12–14% compared to the original MK-40. This may be due to a decrease in the proportion of macropores as a result of a layer of a homogeneous film covering the surface. This is confirmed by the results of studying the structure of the membrane by the method of standard contact porosimetry.

**Figure 10:**
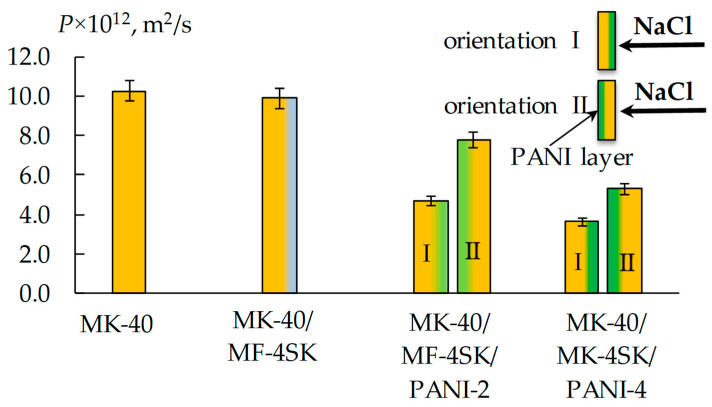
Integral coefficient of diffusion permeability of the initial and modified MK-40 membranes in 0.5 mol/L NaCl solution.

The diffusion permeability of MK-40/MF-4SK/PANI-2 and MK-40/MF-4SK/PANI-4 samples also decreases, and the effect intensifies with increasing modification time from 20–50% for 2 h to 40–60% for 4 h modifier synthesis (Figure 10). An asymmetry in the diffusion permeability for both MK-40/MF-4SK/PANI membranes was found. It consists of a significant decrease of 30 and 40% of the diffusion permeability coefficient in the case of the orientation of the membrane with the modified surface to the salt flow compared to the reverse orientation.

**Figure 11:**
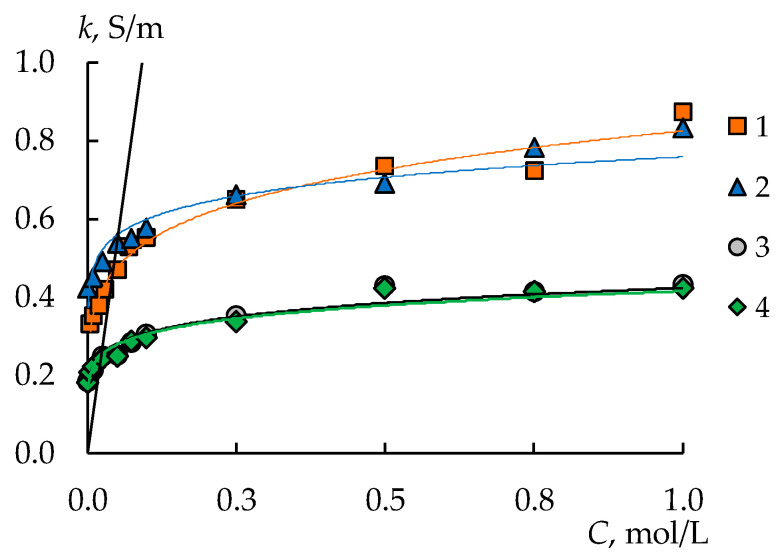
Concentration dependence of specific conductivity in NaCl solution for MK-40 (1), MK-40/MF-4SK (2), MK-40/MF-4SK/PANI-2 (3), and MK-40/MF-4SK/PANI-4 (4). The black line is the NaCl solution conductivity.

The application of the MF-4SK layer on the surface of the MK-40 membrane has almost no impact on the specific conductivity (Figure 11, curves 1–2). This is due to the small amount of MF-4SK. However, the synthesis of PANI leads to a decrease in the specific conductivity of the membranes by 35% for both modified membranes compared to the original membrane (Figure 11, curves 3–4).

In the literature [99], the results of a study of MK-40/PANI composites obtained under similar conditions, but without an MF-4SK layer on a surface modified with PANI (MK-40/PANI-2 and MK-40/PANI-4), are presented. The specific conductivity of the MK-40/PANI-2 and MK-40/PANI-4 membranes in an NaCl solution remains comparable to the specific conductivity of the initial membrane. The diffusion permeability of MK-40/PANI-2 membranes increases by almost three times compared with the initial values. An increase in the PANI synthesis time to 4 h leads to a decrease in the diffusion permeability compared to MK-40/PANI-2. However, the values of the diffusion permeability coefficient of the modified MK-40/PANI-4 sample remain higher than that of the initial membrane. These results differ significantly from those obtained in this work, which is due to the appearance of a layer of a homogeneous MF-4SK membrane on the MK-40 membrane. It is assumed that, as expected in this case, a PANI barrier layer is formed on the entire surface of the MK-40 membrane.

### 3.3. Selectivity of the Bilayer Membranes

**Figure 12:**
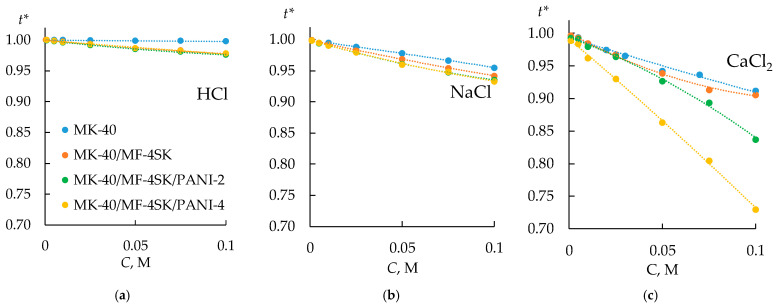
The transport numbers of counterions in the membranes in HCl (**a**), NaCl (**b**), and CaCl_2_ (**c**) solutions.

Based on the obtained concentration dependences of the electrical conductivity of the initial and modified membranes in HCl, NaCl, and CaCl_2_ solutions, the transport numbers of counterions in the membrane (t*) were estimated within the extended three-wire model [102]. The procedure for calculating t* values is described in Appendix A. The obtained concentration dependencies of the transport numbers of counterions in the membrane are shown in Figure 12.

The modification of bilayer membranes with PANI leads to a slight decrease in their selectivity in a solution of NaCl and HCl. However, in a solution of CaCl_2_, the transport numbers decrease by 6–8% for the MK-40/MF-4SK/PANI-2 sample and even more significantly for the MK-40/MF-4SK/PANI-4 sample. A significant decrease in the transport numbers of counterions in the CaCl_2_ solution and the preservation of selectivity at a level close to that of the initial membrane indicates the possible presence of specific selectivity to singly charged cations.

### 3.4. Current–Voltage Curves of the Bilayer Membranes

**Figure 13:**
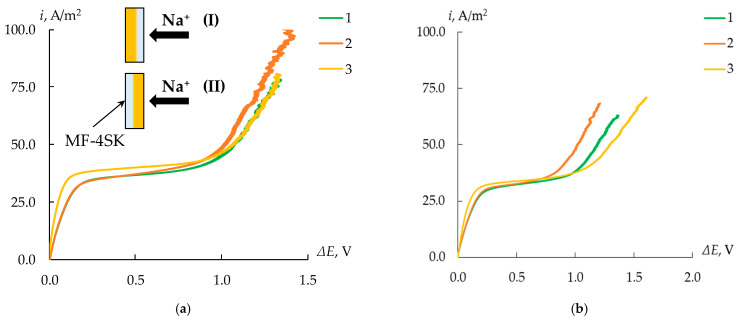
CVCs of the initial MK-40 membrane (3) and the MK-40/MF-4SK membrane (1, 2) when the MF-4SK layer is oriented to the flux of counterions (1, orientation I) and vice versa (2, orientation II) in solutions of 0.05 mol-eq/L NaCl (**a**) and CaCl_2_ (**b**). (I) and (II) here and below represent scheme of the orientation of the modified membrane surface to the counterion flux.

To reveal the effect of the MF-4SK layer on the properties of the MK-40 membrane, the CVCs of the initial membranes and MK-40/MF-4SK membranes were measured (Figure 13). The most noticeable effect was the asymmetry in the length of the limiting current plateau depending on the side of the membrane facing the flow of counterions. Thus, in the case when the surface of the membrane with the MF-4SK layer faces the flow of calcium cations, the length of the limiting current plateau is 17% less than in the reverse orientation. At the same time, for both orientations, the length of the limiting current plateau is less than this value for the initial MK-40 membrane. This may be due to a different pattern of development of conjugated effects of concentration polarization as a result of a change in the microrelief of the membrane surface due to the appearance of a layer of a homogeneous film on one of them.

In general, the low impact of the MF-4SK film on the electrotransport properties of the heterogeneous MK-40 membrane is natural and is due to the surface distribution of the modifier and the small thickness of its layer.

**Figure 14:**
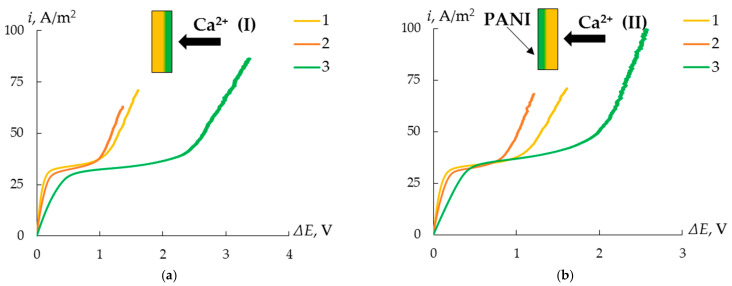
CVCs of the initial MK-40 membrane (1), the MK-40/MF-4SK (2), and the MK-40/MF-4SK/PANI-4 membranes (3) with the modified (**a**) and unmodified (**b**) surface orientation towards counterion flux in a solution of 0.05 mol-eq/L CaCl_2_.

**Figure 15:**
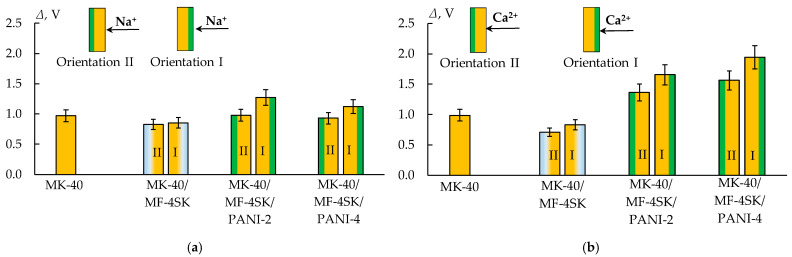
The length of the limiting current plateau of the initial and modified MK-40 membrane in solutions of 0.05 mol/L NaCl (**a**) and 0.05 mol-eq/L CaCl_2_ (**b**).

To assess the possible presence of specific charge selectivity in the obtained membranes, their CVCs were measured (Figure 14). An asymmetry of the CVC of bilayer membranes modified with PANI was found. It consists of an increase in the length of the limiting current plateau when the composite is oriented with the modified side to the counterion flux compared to the reverse orientation by 25–30%, regardless of the membrane modification time (Figure 15).

For the composite with the longest PANI synthesis time (4 h), an insignificant decrease of about 8% in the limiting current density is observed when the modified side is oriented towards the counterion flux (Table 3).

The change in the slope of the ohmic section (Δ*i*_ohm_/Δ*E*_ohm_) of the CVC is coherent with the data on the specific conductivity of membranes: for bilayer membranes, the lowest values of the specific conductivity of the electromembrane system are observed. The traditional dependence of the limiting current density and the slope of the ohmic section on the nature of the electrolyte is also observed: the limiting current density and the slope of the ohmic section decrease in the series HCl > NaCl > CaCl_2_. This is due to the similar value of the diffusion coefficients of electrolytes and the mobility of the corresponding counterions in solution.

## 4. Conclusions

A decrease in the diffusion permeability and specific conductivity of the membranes was found after their modification with PANI. This allows us to conclude that a PANI barrier layer is formed on the membrane surface. The selectivity of the heterogeneous cation-exchange membranes MK-40/MF-4SK/PANI significantly decreases in calcium chloride solution but hardly changes in hydrochloric acid and sodium chloride solutions, compared to the initial MK-40. This indicates the appearance of charge selectivity for modified membranes. However, the detected slight asymmetry in the shape and parameters of the CVC is insignificant and does not indicate the presence of the specific selectivity of the obtained bilayer membranes to singly charged cations. This might be caused by several reasons. The MF-4SK layer on the MK-40 surface may have defects due to the fact that these materials have significant differences in swelling coefficient, which might lead to partial delamination or cracking of the film during the pretreatment of the MK-40/MF-4SK membrane. It is also possible that an insufficiently dense PANI layer is formed. Both of these reasons can lead to the fact that large defects (macropores) remain, and ions are transferred through them, similar to the initial membrane.

## Figures and Tables

**Table 1:** The ion exchange capacity and thickness (*l*) of the initial and the modified membranes.

Membrane	Q, mmol-eq/g	*l*, μm
MK-40	1.6 ± 0.1	560 ± 10
MK-40/MF-4SK	1.7 ± 0.2	550 ± 20
MK-40/MF-4SK/PANI-2	1.6 ± 0.1	550 ± 10
MK-40/MF-4SK/PANI-4	1.6 ± 0.1	540 ± 15

**Table 2:** Physicochemical characteristics of initial and modified membranes.

Membrane	Counterion	*V*_0_, cm^3^/g_dry_	$\frac{V_{\mathrm{micro}}}{V_{0}}$	$\frac{V_{\mathrm{macro}}}{V_{\mathrm{sw}.m}}$	*S*, m^2^/g
MK-40/MF-4SK	H^+^	0.771	0.34	0.31	400
MK-40/MF-4SK/PANI-2		0.608	0.43	0.24	386
MK-40/MF-4SK/PANI-4		0.560	0.49	0.20	375
MK-40/MF-4SK	Ca^2+^	0.747	0.34	0.31	402
MK-40/MF-4SK/PANI-2		0.531	0.47	0.21	420
MK-40/MF-4SK/PANI-4		0.541	0.47	0.21	438

**Table 3:** CVC parameters of the initial and modified MK-40 membranes in solutions of various electrolytes with a concentration of 0.05 mol-eq/L. The bilayer membrane orientation I is the orientation of the modified surface toward counterion flux, and the bilayer membrane orientation II is the opposite orientation.

Membrane	*i*_lim_, A/m^2^		Δ, V		Δ*i*_ohm_/Δ*E*_ohm_	
	Bilayer Membrane Orientation					
	I	II	I	II	I	II
	NaCl					
MK-40	37.2 ± 0.4		0.97 ± 0.02		348 ± 73	
MK-40/MF-4SK	34 ± 1	32.9 ± 0.2	0.85 ± 0.07	0.83 ± 0.03	227 ± 18	229 ± 5
MK-40/MF-4SK/PANI-2	40 ± 1	36 ± 1	1.27 ± 0.07	0.98 ± 0.08	171 ± 12	180 ± 3
MK-40/MF-4SK_PANI-4	36 ± 1	38 ± 2	1.12 ± 0.05	0.93 ± 0.09	191 ± 6	201 ± 7
	HCl					
MK-40	173 ± 11		0.96 ± 0.04		1545 ± 106	
MK-40/MF-4SK	171 ± 18	142 ± 29	0.98 ± 0.07	0.80 ± 0.05	1692.7 ± 121.4	1221 ± 158
MK-40/MF-4SK/PANI-2	165 ± 26	160 ± 5	3.8 ± 0.3	1.8 ± 0.1	1079.8 ± 142.6	1159 ± 90
MK-40/MF-4SK_PANI-4	166 ± 3	184 ± 6	1.6 ± 0.2	1.75 ± 0.3	1350.9 ± 250.2	1250 ± 232
	CaCl_2_					
MK-40	31.7 ± 0.2		0.99 ± 0.01		240.4 ± 14.3	
MK-40/MF-4SK	29.7 ± 0.3	29.7 ± 0.4	0.83 ± 0.01	0.71 ± 0.01	159.8 ± 3.2	168 ± 3
MK-40/MF-4SK/PANI-2	30.2 ± 0.4	30.2 ± 0.4	1.66 ± 0.01	1.4 ± 0.1	36.0 ± 0.5	57 ± 6
MK-40/MF-4SK_PANI-4	30 ± 1	32.7 ± 0.7	1.9 ± 0.2	1.6 ± 0.1	66.1 ± 2.8	81 ± 3

**Figure A1:**
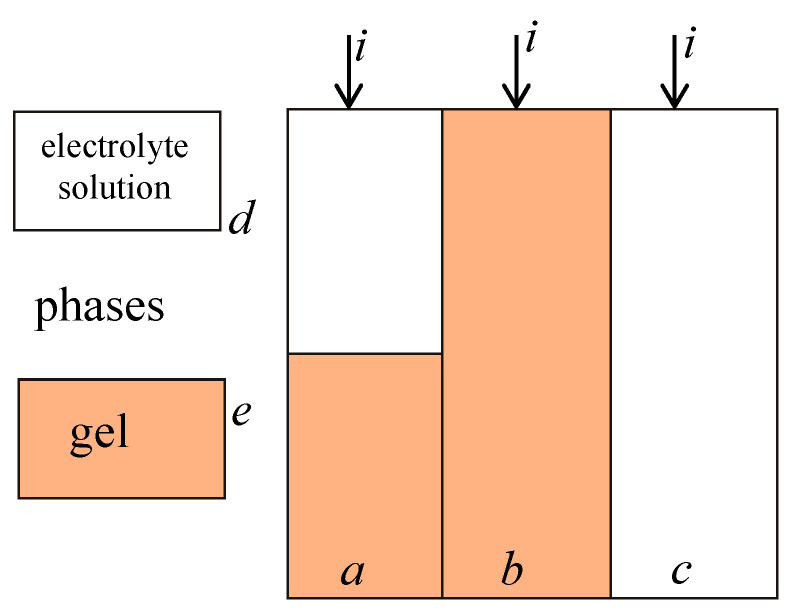
Schematic diagram of the three-wire model of an ion exchange membrane [102]. *i* is current, *a*, *b* and *c* values are current fractions passing through mixed, gel and solution channels, *d* and *e* are fractions of solution and gel phases in mixed channel.

## Data Availability

The data presented in this study are available on request from the corresponding author.

## Appendix A

### Appendix A.1. Extended Three-Wire Model of Ion Exchange Membrane

The membrane structure could be presented as a microheterogeneous system containing two conducting phases. The first phase includes the swollen polymer, i.e., polymer chains, fixed groups, and bounded water, and has unipolar conductivity. The second phase is the internal “free” equilibrium solution with bipolar conductivity similar to the external solution. They do not have a definite interface boundary and represent themselves as. pseudo-phases with chaotic mutual spatial orientation. According to the extended tree-wire model of the ion exchange membrane, the current paths pass through three parallel channels: a gel channel, a channel of intergel solution, and a mixed channel with an interchange of gel and solution phases (Figure A1).

The conductivity of such a system could be described as follows:

(A1) $$K_{m}=\frac{aK_{d}}{e+dK_{d}}+bK_{d}+c,$$

(A2) $$b=f_{1}^{1/\alpha },$$

(A3) $$c=f_{2}^{1/\alpha },$$

(A4) $$a=1-f_{2}^{1/\alpha }-f_{1}^{1/\alpha },$$

(A5) $$d=1-(f_{1}-b)/a,$$

(A6) $$e=\left(f_{1}-b\right)/a,$$

(A7) a+b+c=1,

(A8) d+e=1,

*f*_1_ + *f*_2_ = 1, (A9)

where *K_m_* and *K_d_* are dimensionless conductivities of the membrane and its gel phase; $K_{m}=\frac{k}{k_{\mathrm{sol}}}$, $K_{d}=\frac{k_{\mathrm{iso}}}{k_{\mathrm{sol}}}$, *a*, *b*, and *c* are fractions of current passing through mixed, gel, and solution channels; *d* and *e* are fractions of solution and gel in a mixed channel; *f*_1_ and *f*_2_ are volume fractions of gel and intergel pseudo-phases; *k*_iso_ and *k*_sol_ are conductivities of the membrane gel phase and equilibrium solution; and α is a structural parameter characterizing the spatial orientation of the pseudo-phases inside the membrane (*α* could possess a value in the range from −1 to 1 corresponding to the serial and parallel orientation of conducting phases correspondingly). Considering the gel phase is ideally selective, i.e., the counterion transport number in the gel phase is equal to 1, the counterion transport number in the membrane could be determined as follows:

(A10) $$t^{*}=1-t_{\text{-}}\frac{c}{K_{m}}$$

where *t_-_* is the co-ion transport number in the solution.

### Appendix A.2. The Procedure of t* Calculation

Firstly, the *f*_1_ and α are varied in a range of 0.5–1 and −1–1, respectively. The exact values of *f*_1_ and α are determined by the minimization of the square root deviation of the calculated *K_m_—K_d_* curve by Equations (A1)–(A9) from the experimental one. The *c* parameter is calculated by Equation (A3). The *t** value is determined using Equation (A10).
